# Recombinant amyloid beta-peptide production by coexpression with an affibody ligand

**DOI:** 10.1186/1472-6750-8-82

**Published:** 2008-10-30

**Authors:** Bertil Macao, Wolfgang Hoyer, Anders Sandberg, Ann-Christin Brorsson, Christopher M Dobson, Torleif Härd

**Affiliations:** 1 Department of Medical Biochemistry, University of Gothenburg, PO Box 440, SE-405 30 Göeborg, Sweden; 2 Department of Chemistry, University of Cambridge, Lensfield Road, Cambridge CB2 1EW, UK; 3 The Swedish NMR Centre, University of Gothenburg, Box 465, SE-405 30 Göeborg, Sweden

## Abstract

**Background:**

Oligomeric and fibrillar aggregates of the amyloid β-peptide (Aβ) have been implicated in the pathogenesis of Alzheimer's disease (AD). The characterization of Aβ assemblies is essential for the elucidation of the mechanisms of Aβ neurotoxicity, but requires large quantities of pure peptide. Here we describe a novel approach to the recombinant production of Aβ. The method is based on the coexpression of the affibody protein Z_Aβ3_, a selected affinity ligand derived from the Z domain three-helix bundle scaffold. Z_Aβ3 _binds to the amyloidogenic central and C-terminal part of Aβ with nanomolar affinity and consequently inhibits aggregation.

**Results:**

Coexpression of Z_Aβ3 _affords the overexpression of both major Aβ isoforms, Aβ(1–40) and Aβ(1–42), yielding 4 or 3 mg, respectively, of pure ^15^N-labeled peptide per liter of culture. The method does not rely on a protein-fusion or -tag and thus does not require a cleavage reaction. The purified peptides were characterized by NMR, circular dichroism, SDS-PAGE and size exclusion chromatography, and their aggregation propensities were assessed by thioflavin T fluorescence and electron microscopy. The data coincide with those reported previously for monomeric, largely unstructured Aβ. Z_Aβ3 _coexpression moreover permits the recombinant production of Aβ(1–42) carrying the Arctic (E22G) mutation, which causes early onset familial AD. Aβ(1–42)E22G is obtained in predominantly monomeric form and suitable, e.g., for NMR studies.

**Conclusion:**

The coexpression of an engineered aggregation-inhibiting binding protein offers a novel route to the recombinant production of amyloidogenic Aβ peptides that can be advantageously employed to study the molecular basis of AD. The presented expression system is the first for which expression and purification of the aggregation-prone Arctic variant (E22G) of Aβ(1–42) is reported.

## Background

Alzheimer's disease (AD) is the most common neurodegenerative disorder, currently afflicting about 20 million people worldwide, with increasing prevalence in an ageing society [1]. AD is characterized by large extracellular deposits of senile plaques in the brain, consisting of aggregated, fibrillar amyloid β-peptide (Aβ) [2,3]. Extensive evidence supports a critical role of soluble intermediary Aβ oligomers in the induction of synapse dysfunction and neurodegeneration [3-6]. Aβ originates from proteolytic processing of the amyloid precursor protein (APP) [7]. APP is cleaved by the membrane associated β- and γ-secretases that generate a number of differently sized peptides, of which Aβ(1–40) and Aβ(1–42) are most abundant. Aβ(1–42) is considerably more neurotoxic than Aβ(1–40), in agreement with its increased hydrophobicity and tendency to aggregate. Mutations within Aβ are associated with familial AD and cerebral amyloid angiopathy. One example is the Arctic (E22G) mutation, which entails enhanced Aβ protofibril formation and fibrillation and causes typical AD neuropathology [8,9].

Despite the fact that much effort has been put into Aβ-related research, many questions still need to be answered. Most importantly, the precise mechanisms of Aβ toxicity remain to be understood [3]. In this context, an inventory of oligomeric and protofibrillar Aβ species would be desirable, detailing their biophysical properties and contributions to neurodegeneration. The extension and refinement of existing structural data on Aβ oligomers and fibrils [10-12] would help to derive structure-toxicity relationships and thus support AD drug discovery efforts. The accessibility of large amounts of Aβ peptide is a prerequisite for these studies.

The majority of research using Aβ peptides within the areas of biochemistry, biophysics and cell biology is conducted with synthetic peptides. An alternative to chemical synthesis is recombinant expression in *Escherichia coli*, which is advantageous because of its low cost, the fast growth to high expression levels and the availability of established cloning and expression protocols [13]. Recombinant expression is particularly attractive for structural biology projects, as it enables the production of milligram quantities of isotope or seleno-methionine labeled peptide for structure determination by nuclear magnetic resonance (NMR) spectroscopy or x-ray crystallography at reasonable cost.

Prokaryotic expression and purification of highly amyloidogenic peptides such as Aβ has proven difficult due to their small size, their tendency to aggregate and the toxicity of the formed aggregates [14]. Protein fusions, which might protect from proteolysis and enhance solubility, are typically used to tackle these problems [13,15,16]. The expression of Aβ(1–40) or Aβ(1–42) fused to segments of a surface protein from the malaria parasite *Plasmodium falciparum *[17], maltose binding protein [18], ubiquitin [19], GroES-ubiquitin [20], trigger factor-ubiquitin [21], and hen egg white lysozyme [22] has been reported. In order to obtain Aβ unaffected by the tag, its removal by site specific proteolysis is an inevitable additional purification step in all of these cases. The proteolytic cleavage reaction is cost-intensive, requires time-consuming optimization and necessitates post-reaction clean-up, which further reduces the attainable yield.

An alternative method to increase the yield of troublesome target proteins is coexpression with proteins that stabilize the target, assist with its folding, or prevent its aggregation [23]. This technique has permitted heterologous expression of macromolecular complexes, whose components could not be obtained individually [24-27]. Co-overexpression of molecular chaperones can increase the yield of targets to varying extents [28,29].

**Figure 1:**
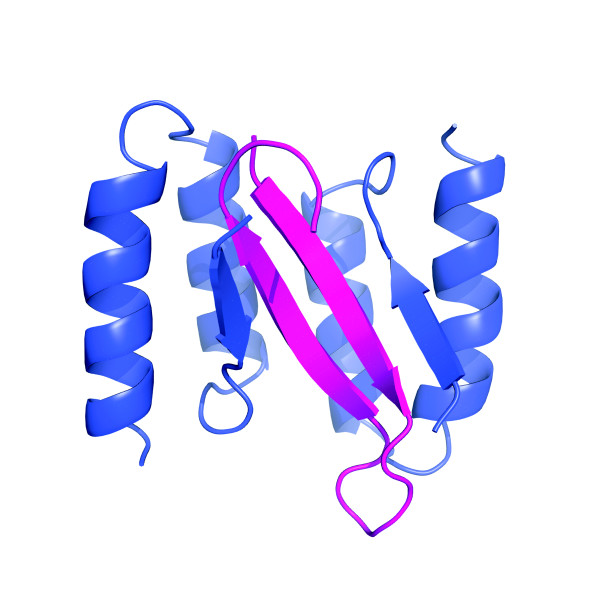
**Structure of the Aβ(1–40):Z_Aβ3 _complex**. Ribbon drawing of the topology of the complex [33]. Aβ(1–40) is shown in magenta, Z_Aβ3 _in blue. The disordered N-termini are not displayed. The image was generated using PyMOL (DeLano Scientific).

Here we present a novel approach to the recombinant production of amyloidogenic Aβ peptides. Aβ is obtained by coexpression with an engineered binding protein that specifically binds and stabilizes the monomeric peptide. The binding protein, termed Z_Aβ3_, belongs to the class of affibody affinity ligands [30,31]. Affibody proteins have found applications in biotechnology, biochemical assays, disease diagnosis and therapy [31]. They are selected by phage display from libraries based on the 58 amino acid three-helix bundle scaffold of the Z domain derived from staphylococcal protein A [32]. Z_Aβ3 _is a disulfide-linked homodimer of affibody subunits that binds monomeric Aβ with nanomolar affinity [33] (Figure 1). In contrast to the majority of Aβ-antibodies [34], Z_Aβ3 _targets the highly amyloidogenic central and C-terminal part of Aβ (residues 17–36). This region adopts a β-hairpin conformation upon binding and is buried within a hydrophobic tunnel-like cavity formed by Z_Aβ3_. Consequently, Aβ oligomerization and fibrillation are inhibited by stoichiometric concentrations of Z_Aβ3 _[33].

The concept of producing Aβ by recombinant coexpression with Z_Aβ3 _offers several potential advantages: (*i*) Binding of the coexpressed Z_Aβ3 _to the amyloidogenic sequence region of Aβ could retain the peptide in a monomeric state during expression and the initial purification steps, thereby preventing any cell toxicity exerted by aggregates and facilitating purification. (*ii*) Aβ is largely unfolded in its unbound monomeric state [35], and the complex might therefore protect the peptide from degradation. (*iii*) Both coexpression of auxiliary proteins and protein fusions impose additional metabolic burden on host cells, but the small size of the affibody scaffold limits this burden, which is especially important when short peptides such as Aβ are to be expressed.

In the present implementation of the Aβ:Z_Aβ3 _coexpression system, Aβ is expressed tag-less, offering a particularly facile route to obtain pure peptide. As a consequence, a methionine resulting from the obligatory translation start codon is obtained N-terminal of Aβ. The resulting peptide will thus be referred to as MAβ below.

We report the expression and purification of ^15^N-labeled MAβ(1–40), MAβ(1–42) and MAβ(1–42)E22G by Z_Aβ3 _coexpression. The method yields pure, fibrillation-competent, monomeric peptides with conformational properties and aggregation propensities indistinguishable from those of the respective Aβ peptides.

## Results

### Expression and purification of MAβ peptides

**Figure 2:**
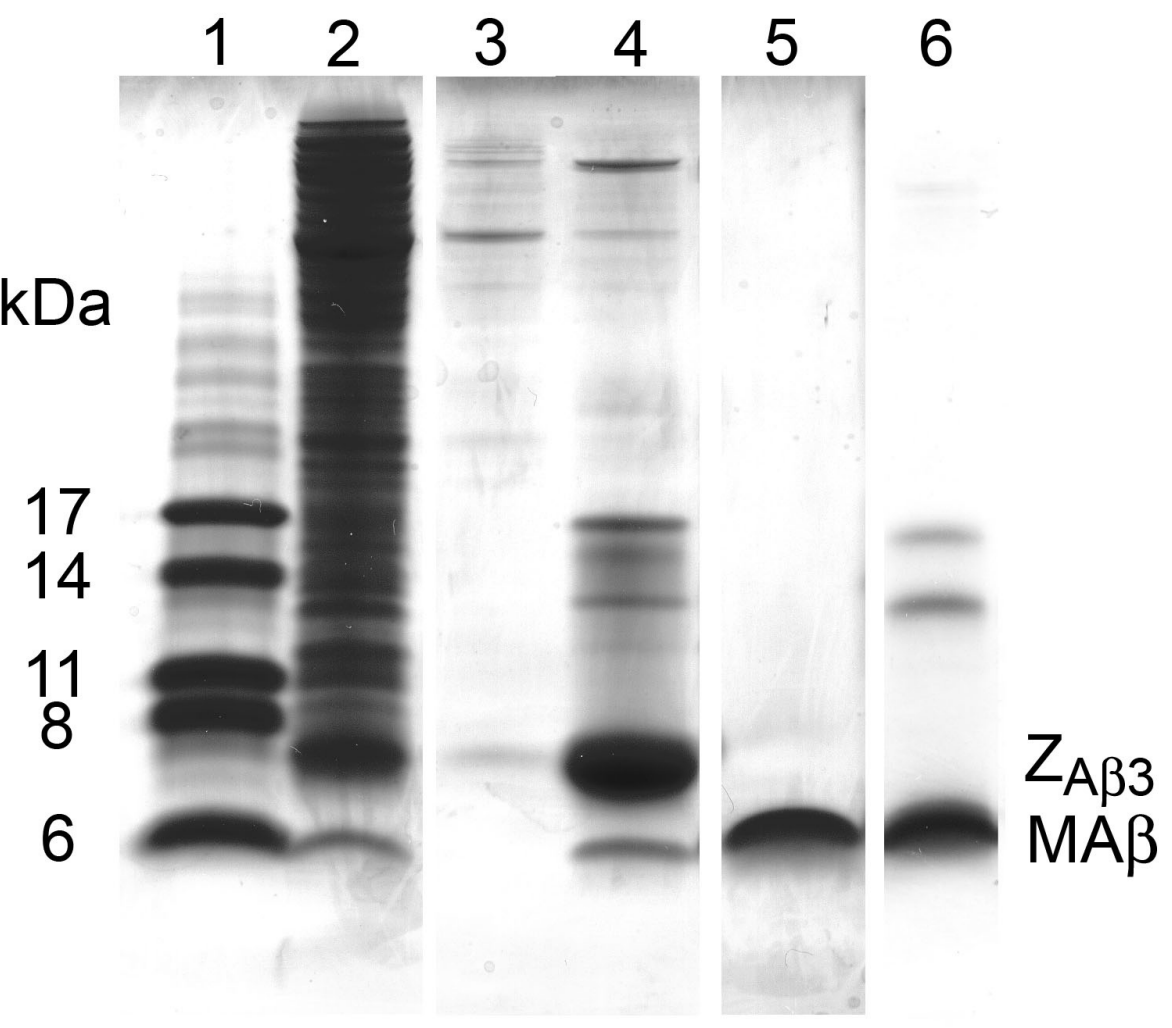
**Purification of MAβ peptides**. SDS-PAGE on a 16.5% Tris-Tricine gel at 4°C. Lane 1: Marker. 2: Cell lysate after MAβ(1–40) coexpression. 3: IMAC wash fraction (10 mM imidazole). 4: IMAC eluate after addition of 150 mM imidazole, demonstrating the effective capture of the Z_Aβ3_:MAβ(1–40) complex. 5: Purified MAβ(1–40). 6: Purified MAβ(1–42). Samples were incubated for 2 min at 95°C prior to loading.

A double cistronic coexpression vector based on the bacterial expression vector pACYCDuet-1 (Novagen) was constructed. The coexpression vector contains the genes for MAβ [MAβ(1–40), MAβ(1–42), or MAβ(1–42)E22G] and (His)_6_-tagged Z_Aβ3 _in the following order: T7 promoter-1 – MAβ – T7promoter-2 – (His)_6_Z_Aβ3 _– T7 terminator. MAβ is effectively overexpressed and obtained in the soluble fraction of cell lysates, indicating that its complex with the disulfide-linked Z_Aβ3 _dimer is formed and stable in the *E. coli *cytosol (Figure 2, lane 2).

**Figure 3:**
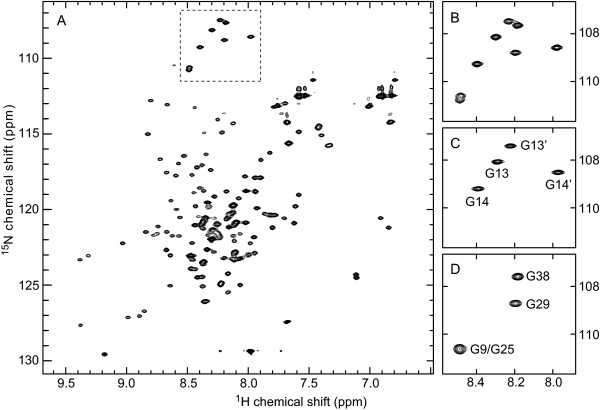
**NMR spectroscopy of purified MAβ:Z_Aβ3 _complex**. (A) ^15^N HSQC NMR spectrum of the ^15^N-labeled MAβ(1–40):Z_Aβ3 _complex. The complex was obtained from coexpression and purified by IMAC (elution of the intact complex with 150 mM imidazole) and SEC. (B)-(D) Comparison of the glycine region (B; boxed area in panel A) with corresponding regions of ^15^N HSQC spectra of Aβ(1–40):Z_Aβ3 _samples in which either Z_Aβ3 _(C) or Aβ(1–40) (D) are ^15^N-labeled. Sequential assignments of glycine resonances for the two Z_Aβ3 _subunits and bound Aβ(1–40) are given in panels C and D, respectively. The occurrence of resonances at identical chemical shifts in complexes of Z_Aβ3 _with recombinant MAβ(1–40) and native Aβ(1–40) peptides indicates that the structures of the two complexes are the same. NMR was measured at 25°C at 800 MHz (A, B and D) or 900 MHz (C) on samples containing 160 μM (A, B), 450 μM (C) or 400 μM (D) complex in 20 mM potassium (A, B) or sodium (C, D) phosphate, with 0.1% azide and 10% D_2_O at pH 7.2.

In the present system, the auxiliary protein Z_Aβ3 _is (His)_6_-tagged but not the target peptide, permitting the purification of tag-free MAβ without a cleavage reaction. MAβ is captured in complex with Z_Aβ3 _by immobilized metal ion affinity chromatography (IMAC) (Figure 2, lane 4), demonstrating that the MAβ:Z_Aβ3 _complex remains stable during the initial purification steps. Resonances in the ^15^N heteronuclear single quantum correlation (HSQC) NMR spectrum of the coexpressed MAβ(1–40):Z_Aβ3 _complex coincide with those of the native Aβ(1–40):Z_Aβ3 _complex, indicating that their structures are identical (Figure 3). MAβ is not detected in the IMAC wash fraction (Fig. 2, lane 3). Dissociation of the complex during IMAC is consequently not limiting the peptide yield.

Separation of the MAβ:Z_Aβ3 _complex is achieved by IMAC under denaturing conditions. Pure monomeric MAβ is subsequently obtained by application of the denatured peptide to size exclusion chromatography (SEC) using native running buffer, e.g., 20 mM sodium phosphate, 50 mM sodium chloride, pH 7.2.

SDS-PAGE shows a single band corresponding to the monomeric peptide in the case of purified MAβ(1–40), whereas two additional bands at higher molecular weight, approximately at 12 and 15 kDa, are observed for MAβ(1–42) (Figure 2). These bands have been observed before and have been attributed to the SDS-induced formation of Aβ(1–42) oligomers [4,36]. Mass spectrometry confirmed that the bands consist of MAβ(1–42).

The peptide yield from a 1 L culture was 4 mg of MAβ(1–40) or 3 mg of MAβ(1–42). Purification of Z_Aβ3 _from an MAβ(1–40) coexpression culture gave 23 mg of the dimeric protein per 1 L of culture, indicating that ~60% of the expressed Z_Aβ3 _was in complex with MAβ(1–40), whereas the rest remained unbound.

### Comparison of MAβ with Aβ

**Figure 4:**
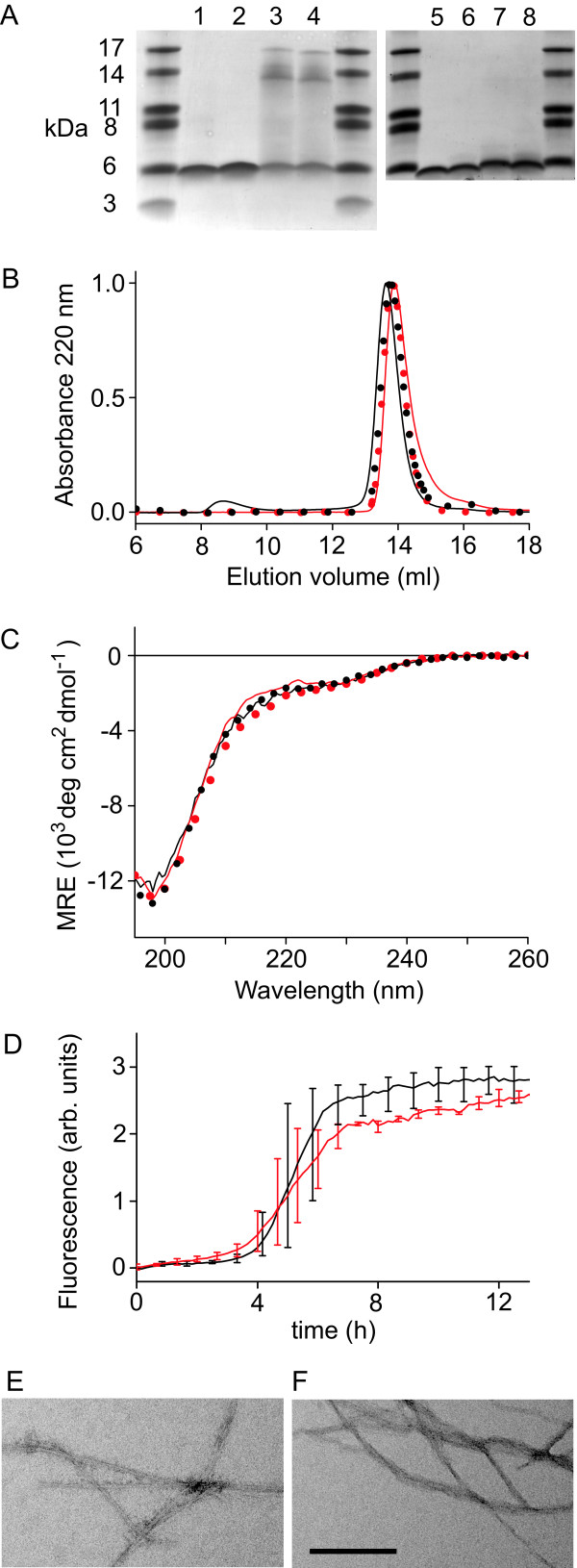
**Comparison of MAβ with Aβ**. (A) SDS-PAGE using 16.5% Tris-Tricine gels at 21°C (lanes 1–4) or 4°C (lanes 5–8). Lanes 1 and 5: MAβ(1–40). 2 and 6: Aβ(1–40). 3 and 7: MAβ(1–42). 4 and 8: Aβ(1–42). Unlabeled lanes contain marker. Samples were incubated for 2 min at 95°C prior to loading. (B) Elution profiles of analytical SEC of MAβ(1–40) (red line), Aβ(1–40) (red circles), MAβ(1–42) (black line), Aβ(1–42) (black circles). (C) Far-UV CD spectra of MAβ(1–40) (red line), Aβ(1–40) (red circles), MAβ(1–42) (black line), Aβ(1–42) (black circles). (D) Kinetics of amyloid fibril formation of MAβ(1–42) (red) and Aβ(1–42) (black) monitored by thioflavin T fluorescence. The average of 3 time traces is shown with error bars representing the maximal and minimal values. The peptides were used at 25 μM in 20 mM sodium phosphate, 50 mM sodium chloride, 10 μM thioflavin T, pH 7.2. Temperature, 37°C. (E) and (F) Electron micrographs of amyloid fibrils formed by MAβ(1–42) (E) and Aβ(1–42) (F). Scale bar, 200 nm.

Several different techniques were used to establish that MAβ and Aβ possess identical conformational properties and aggregation propensities. MAβ and Aβ are indistinguishable by SDS-PAGE (Figure 4A). The extent of SDS-induced oligomer formation of MAβ(1–42) is the same as for Aβ(1–42) and increases with temperature.

In SEC, which separates molecules based on their hydrodynamic volume, very similar elution volumes are obtained for MAβ and Aβ (Figure 4B). The elution volumes correspond to a molecular weight of ~11 kDa on a scale calibrated with globular protein standards, in agreement with previous SEC studies [37]. The high apparent molecular weight (the nominal weights of the Aβ and MAβ peptides are in the range of 4.3 to 4.7 kDa) is expected for a peptide that is disordered and consequently has a larger hydrodynamic volume than a globular protein of the same molecular weight.

The secondary structure content was analyzed by circular dichroism (CD) spectroscopy (Figure 4C). Far-UV CD spectra of MAβ conformed to those of Aβ, featuring a minimum at ~198 nm that is characteristic of the predominantly random coil conformation detected in non-aggregated Aβ peptides [38,39].

**Figure 5:**
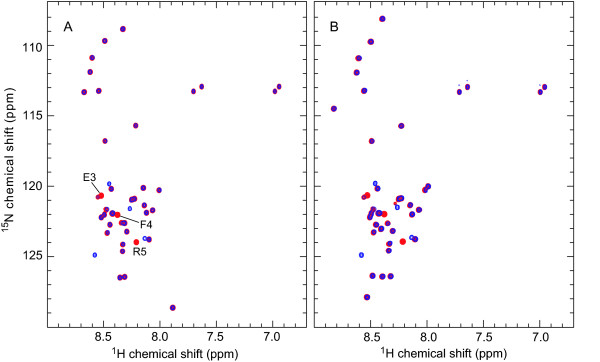
**NMR spectroscopy of MAβ**. ^15^N HSQC NMR spectra of MAβ(1–40) (A, blue), Aβ(1–40) (A, red), MAβ(1–42) (B, blue) and Aβ(1–42) (B, red) at 5°C at 800 MHz. The spectra illustrate the purity of the recombinantly expressed peptides. The chemical shifts and sharp NMR resonances indicate that the peptides exist in disordered monomeric conformations. Resonances of Aβ(1–40) that are displaced as a consequence of the presence of the N-terminal methionine in MAβ(1–40) are indicated. Assignments were obtained from literature spectra [35,52].

The ^15^N HSQC NMR spectra of MAβ(1–40) and MAβ(1–42) strongly resemble those of Aβ(1–40) and Aβ(1–42), respectively (Figure 5). The large majority of Aβ backbone amide resonances are recovered at identical positions in the MAβ spectra. Differences in chemical shifts are only observed for residues N-terminal of Arg5. Such local shift changes are a mandatory consequence of the modification of the peptide sequence, in this case with the N-terminal methionine, and reflect local changes in the electronic environment. However, the chemical shift differences do not demonstrate any change in peptide conformation. The ^15^N HSQC spectra prove that Met35 is unoxidized, by comparison with reference spectra for Aβ(1–40) and Aβ(1–42) [35], in agreement with the mass spectrometry results.

The enhanced fluorescence emission of the dye thioflavin T upon binding to amyloid fibrils is frequently used to monitor fibrillation [40]. The fibrillation kinetics of Aβ(1–42) and MAβ(1–42) are identical within the error of the experiment (Figure 4D). The presence of amyloid fibrils in the final steady-state of fibrillation was confirmed by electron microscopy (Figure 4E and 4F).

### Production and characterization of MAβ(1–42)E22G

**Figure 6:**
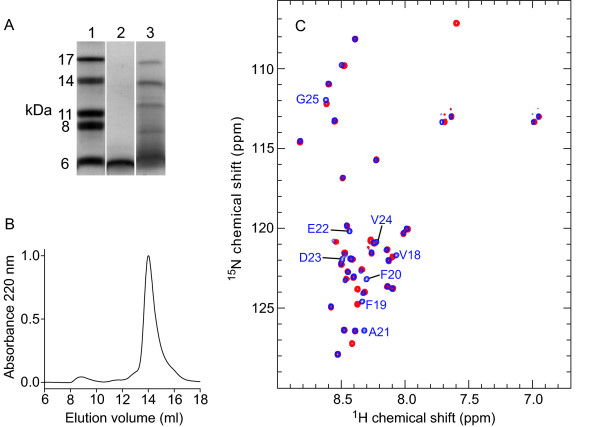
**Recombinant expression of MAβ(1–42)E22G**. (A) SDS-PAGE using a 16.5% Tris-Tricine gel at 4°C. Lane 1: Marker. 2: MAβ(1–42). 3: MAβ(1–42)E22G. (B) Elution profile of analytical SEC of purified MAβ(1–42)E22G. (C) ^15^N HSQC NMR spectra of MAβ(1–42) (blue) and MAβ(1–42)E22G (red) at 5°C at 800 MHz. Resonances of MAβ(1–42) that are lost or displaced as a consequence of the E22G mutation are indicated. Assignments were obtained from literature spectra [35,52].

The Arctic mutant of Aβ is a particularly interesting variant inasmuch as it links an increased tendency for protofibril formation and fibrillation to early onset familial AD [8,9]. To our knowledge, no protocol for the recombinant production of Aβ(1–42)E22G has been reported to date, possibly due to the extreme aggregation propensity of this peptide variant. Coexpression of Z_Aβ3 _permitted the production of MAβ(1–42)E22G with a yield of 1 mg from a 1 L culture. SDS-PAGE demonstrates increased oligomerization of MAβ(1–42)E22G compared to MAβ(1–42) (Figure 6A). The major fraction of purified MAβ(1–42)E22G is present in monomeric form as evidenced by SEC, which gives an elution volume of ~14 mL (Figure 6B), similar to that of monomeric MAβ(1–40) and MAβ(1–42) (Figure 4B). Recombinant MAβ(1–42)E22G can be employed, e.g., for NMR spectroscopy. The ^15^N HSQC NMR spectrum of MAβ(1–42)E22G is displayed in Figure 6C. As expected, the resonances of backbone amides in the vicinity of residue 22 are affected by the E22G mutation due to the removal of one negative charge. However, the changes are not large and the E22G mutant is also disordered in its monomeric state.

## Discussion

We have shown that coexpression of the Z_Aβ3 _affibody protein enables recombinant production of MAβ peptides. Z_Aβ3 _binds to the amyloidogenic, hydrophobic central and C-terminal region of Aβ and thus prevents peptide aggregation and potential detrimental effects on cellular homeostasis. MAβ is released from Z_Aβ3 _only after an initial purification of the complex has been achieved, limiting the potential for adverse effects during peptide preparation.

Coexpression is particularly profitable for the production of unstructured proteins that exhibit folding coupled to binding to the coexpressed partner [41]. The Aβ:Z_Aβ3 _interaction is characterized by extensive coupled folding and binding of both binding partners [33,42]. In addition to increasing the thermodynamic stability of the complex's constituents, coupled folding-binding presumably also reduces their susceptibility to proteolytic degradation.

Just as protein fusion tags preferably should be short, coexpression systems profit from a small size of the auxiliary protein, as this limits the metabolic burden. The affibody scaffold used for engineering Z_Aβ3 _is particularly small and a large fraction of the surface area is involved in the interaction with the target [31,43]. Affibody ligands therefore represent promising auxiliary proteins for the development of coexpression systems, as exemplified in this study.

In contrast to previously published methods for recombinant expression of Aβ [17-22,44], the present system does not require a cleavage reaction. The reported cleavage reactions are time-consuming and/or expose the peptide to elevated temperature (typically, 37°C), which is detrimental to the production of aggregation-prone peptides [20]. All of these methods moreover include a reverse phase chromatography step, which necessitates thorough subsequent disaggregation of Aβ peptides [38,45,46]. The coexpression system avoids acidic pH and organic solvents, and non-aggregated MAβ in buffered aqueous solution is obtained directly from size exclusion chromatography. Some of the expression methods previously reported have further specific disadvantages compared to the present system: The cleavage reaction causes microheterogeneities [17], Met35 is oxidized [17], the peptide yield is lower [22], or the peptide contains additional residues at the N-terminus [44,47]. In the case of the maltose binding protein fusion, a far greater peptide yield has been achieved [18]. This can in part be explained by the use of a fermentation system, which allows higher cell densities to be reached, frequently resulting in >10-fold the amount of purified protein in comparison to shake flask cultures [48,49]. The maltose binding protein fusion has only been used for production of the less aggregation-prone variant Aβ(1–40) and provides peptide that aggregates into predominantly non-fibrillar structures [50,51].

The MAβ peptides contain a methionine N-terminal of the Aβ sequence, originating from the translation start codon. However, according to the biochemical and biophysical analysis of the peptides by SDS-PAGE, SEC, NMR and CD spectroscopy, and to their aggregation propensity and aggregate morphology, the MAβ peptides faithfully recapitulate the properties of Aβ. The present coexpression system could be adapted to provide the peptide free of the additional N-terminal methionine by expressing a suitably tagged Aβ peptide that can be cleaved to yield the native N-terminus. This would however delimit the ease and cost-effectiveness of the method. Alternatively, secretion signal sequences could be added to both Aβ and affibody. These would be expected to be proteolyzed upon secretion and Aβ would thereby obtain its native N-terminus.

The ^15^N HSQC NMR spectra of MAβ and Aβ presented here superimpose on those reported previously in studies that thoroughly characterized Aβ as largely monomeric under the applied experimental conditions [35,52]. This is in agreement with the observation that purified MAβ and Aβ adopt a predominantly random coil conformation (Figure 4C). We conclude that MAβ is purified in non-aggregated, monomeric form and applicable as starting material for the investigation of fibril (Figure 4D) and oligomer formation. The potential to obtain labeled peptide makes MAβ suitable for structural studies by, e.g., NMR spectroscopy. The coexpression system is compatible with the overexpression of the highly aggregation prone Arctic mutant of MAβ(1–42) and can therefore be utilized to analyze the structural consequences of this and presumably other disease-related mutations of full-length Aβ(1–42). Previous systematic NMR studies on clinically relevant amino acid substitutions have pointed to a connection between monomer folding and oligomerization propensity, but were limited to decapeptide segments of Aβ [53].

## Conclusion

We have described a recombinant expression system that provides facile access to both major isoforms of the highly amyloidogenic Aβ peptide by coexpression of an engineered aggregation-inhibiting binding protein. The method also allows for the production of the particularly oligomerization and fibrillation-prone Arctic (E22G) mutant of Aβ(1–42). The peptides are obtained in non-aggregated, monomeric form and can be favorably applied for the investigation of Aβ oligomerization and fibrillation, inclusive of structural biology studies.

## Methods

### Cloning

The bacterial expression vector pACYCDuet-1 (Novagen) is designed for the double cistronic coexpression of two target genes and contains two multiple cloning sites (MCS), each of which is preceded by a T7 promoter/lac operator and a ribosome binding site. pACYCDuet-1 encoding Aβ(1–40) with an additional N-terminal methionine, cloned as a NcoI/HindIII fragment at MCS 1, was obtained from GENEART. The expression plasmid pAY 442 encoding (His)_6_-tagged Z_Aβ3 _[30] was digested with NdeI and Bpu1102I (all enzymes supplied by New England Biolabs), followed by insertion of the (His)_6_-Z_Aβ3 _gene into MCS2 of pACYCDuet-1 at the respective restriction sites. The resulting coexpression vector contains the genes in the following order: T7 promoter-1 – MAβ(1–40) – T7promoter-2 – (His)_6_Z_Aβ3 _– T7 terminator. The vectors for coexpression of MAβ(1–42) and MAβ(1–42)E22G were generated by site-directed mutagenesis (Stratagene QuikChange mutagenesis kit) of the MAβ(1–40) or MAβ(1–42) expression clones, respectively.

### Protein expression

BL21(DE3) *E. coli *cells (Novagen) were transformed with the expression vectors and grown for ~16 h at 37°C on LB agar plates containing 34 μg/mL chloramphenicol. Single colonies were picked and grown for ~16 h in 20 mL ^15^N-labeled M9 medium, containing 1 g/L ^15^NH_4_Cl, 2 g/L glucose, 2 mM MgSO_4_, 0.1 mM CaCl_2_, 2 g/L natural ^15^N-Celtone powder (Spectra Stable Isotopes) and 34 μg/mL chloramphenicol. The pre-culture was transferred to 1 L of ^15^N-labeled M9-Celtone medium in a 5 L baffled Erlenmeyer flask. The culture was grown at 37°C with shaking and induced at OD600 ~0.8 by the addition of IPTG to a final concentration of 1 mM. After further growth for 4 hours the cells were harvested and frozen at -20°C. If isotopic labeling was not required, TB medium was used as an alternative to M9-Celtone.

### Purification of MAβ

The cell pellet from 1 L of bacterial culture was thawed in an ice/water bath, resuspended in 15 mL of buffer A (50 mM sodium phosphate, 0.2 M sodium chloride, 1 mM PMSF, pH 7.2) and subjected to three freeze-thaw cycles, followed by sonication according to a standard protocol. The lyzed cells were clarified by centrifugation at 17,000 *g *in a JA 25.50 rotor (Beckman) at 4°C for 30 min.

For capture of the MAβ:Z_Aβ3 _complex by IMAC, the supernatant was added to 10 mL HIS-Select Ni^2+ ^affinity gel (Sigma-Aldrich) equilibrated in buffer A, and the mixture was incubated batch wise on a roller shaker for 20 min at room temperature. Proteins not bound to the resin were separated by centrifugation at 700 *g *for 5 min on a swing-out rotor. The resin was washed twice with 50 mL of buffer A, transferred to a 1.5 cm diameter Econo-Column chromatography column (Bio-Rad Laboratories) and washed with another 50 mL of buffer A and 50 mL of buffer A supplemented with 10 mM imidazole. To separate MAβ from the resin-bound Z_Aβ3_, the drained resin was resuspended in 40 mL of buffer SL (buffer A supplemented with 6 M guanidine hydrochloride (GdmCl), pH 7.2) and incubated for 30 min at room temperature. The denatured MAβ peptide was recovered in the filtrate upon filtration of the resin slurry through the Econo-Column chromatography column.

Native MAβ was obtained by SEC of the denatured MAβ on a Superdex 75 HR 10/300 column (GE Healthcare) equilibrated with 20 mM sodium phosphate, 50 mM sodium chloride, pH7.2. If protein concentration or storage was desired, the pH of the SEC eluate was adjusted to basic pH (~10.5) directly after elution, as basic pH preserves the monomeric state of Aβ and is thus advantageous for stock solutions [38,54]. Concentration of the basic protein solutions was achieved using Vivaspin concentration columns (Sartorius). The identity of the peptides was verified by mass spectrometry (MAβ(1–40), theoretical mass: 4458.2 Da, experimental mass: 4458.1 Da; MAβ(1–42), theoretical mass: 4642.3 Da, experimental mass: 4642.3 Da). Peptide concentrations in solution were measured by UV spectroscopy (ε_280_–ε_300 _= 1424 M^-1 ^cm^-1^).

For analysis of the coexpressed MAβ:Z_Aβ3 _complex, the entire complex (i.e. without separation of the complex's constituents under denaturing conditions) was eluted from the IMAC column with buffer A supplemented with 150 mM imidazole, and subjected to SDS-PAGE (Figure 2, lane 4). NMR measurements on the complex (Figure 3) were carried out after an additional SEC step using a Superdex 75 HR 10/300 column equilibrated with 20 mM potassium phosphate, pH 7.2.

### Aβ peptides

Aβ was obtained from a commercial source (rpeptide). Aβ(1–40) was purchased either NaOH pre-treated or HFIP pre-treated, dissolved in 30 mM ammonium hydroxide to a concentration of 0.5 mM, and diluted into the final experiment buffer. Aβ(1–42) was purchased HFIP pre-treated. To ensure disaggregation of Aβ(1–42), the peptide was dissolved in 6 M GdmCl (buffer SL) and subjected to SEC, using the same conditions as employed in the final step of the MAβ purification protocol.

### Analytical size exclusion chromatography

Peptides at a concentration of 40–100 μM were analyzed on a Superdex 75 HR 10/300 column equilibrated in 20 mM sodium phosphate, 50 mM sodium chloride, pH 7.2. Elution profiles were normalized to unity at maximum absorbance for the purpose of comparison.

### Circular dichroism spectroscopy

Far-UV CD measurements were performed on a JASCO J-810 spectropolarimeter using a 0.1 cm path length cuvette. Peptides were used at concentrations of 20–25 μM in 20 mM phosphate, pH 7.2–7.4. Spectra were recorded at 20°C. Thirty scans were averaged without smoothing and corrected for the buffer spectrum.

### Electron microscopy

Samples were applied to formvar/carbon coated nickel grids, stained with 2% (w/v) uranyl acetate and viewed in a LEO 912 AB Omega transmission electron microscope.

### NMR spectroscopy

NMR was measured using Varian Inova 800 MHz and 900 MHz spectrometers. Samples of the MAβ:Z_Aβ3 _complex prepared after IMAC and SEC purification contained 160 μM complex in 20 mM potassium phosphate, pH 7.2, with 10% D_2_O. Samples of purified MAβ and commercial Aβ peptides contained *ca*. 60 μM ^15^N-labeled peptides in 20 mM sodium phosphate, 50 mM sodium chloride, pH 7.2, with 10% D_2_O. NMR data were processed using NMRpipe [55] and analyzed using CcpNmr Analysis [56].

### Thioflavin T amyloid formation assay

Thioflavin T fluorescence was recorded in 96-well plates (Nunc) using a FLUOstar Optima reader (BMG) equipped with 440 nm excitation and 480 nm emission filters. The samples contained 100 ml of 25 μM MAβ(1–42) or Aβ(1–42) in 20 mM Na-phosphate, 50 mM sodium chloride, pH 7.2, supplemented with 10 μM thioflavin T. Plates were sealed with polyolefin tape (Nunc) and incubated at 37°C. Data points were recorded every 10 min with 50 sec of orbital shaking (width 5 mm) preceding each measurement.

## Authors' contributions

BM carried out the major part of the DNA work and established the purification protocol. WH carried out the major part of the peptide characterization and wrote the final version of the manuscript. AS conceived of the study and participated in establishing the purification protocol and in circular dichroism studies. ACB and CMD participated in designing and performing electron microscopy experiments. TH participated in the design of the study, coordinated it, and participated in the NMR experiments. All authors read and approved the final manuscript.

## References

[B1] Mount C, Downton C (2006). Alzheimer disease: progress or profit?. Nat Med.

[B2] Findeis MA (2007). The role of amyloid β peptide 42 in Alzheimer's disease. Pharmacol Ther.

[B3] Haass C, Selkoe DJ (2007). Soluble protein oligomers in neurodegeneration: lessons from the Alzheimer's amyloid β-peptide. Nat Rev Mol Cell Biol.

[B4] Bitan G, Fradinger EA, Spring SM, Teplow DB (2005). Neurotoxic protein oligomers – what you see is not always what you get. Amyloid.

[B5] Lambert MP, Barlow AK, Chromy BA, Edwards C, Freed R, Liosatos M, Morgan TE, Rozovsky I, Trommer B, Viola KL (1998). Diffusible, nonfibrillar ligands derived from Aβ_1–42 _are potent central nervous system neurotoxins. Proc Natl Acad Sci USA.

[B6] Walsh DM, Klyubin I, Fadeeva JV, Cullen WK, Anwyl R, Wolfe MS, Rowan MJ, Selkoe DJ (2002). Naturally secreted oligomers of amyloid β protein potently inhibit hippocampal long-term potentiation in vivo. Nature.

[B7] Wolfe MS, Guenette SY (2007). APP at a glance. J Cell Sci.

[B8] Johansson AS, Berglind-Dehlin F, Karlsson G, Edwards K, Gellerfors P, Lannfelt L (2006). Physiochemical characterization of the Alzheimer's disease-related peptides Aβ1–42Arctic and Aβ1–42wt. FEBS J.

[B9] Nilsberth C, Westlind-Danielsson A, Eckman CB, Condron MM, Axelman K, Forsell C, Stenh C, Luthman J, Teplow DB, Younkin SG (2001). The 'Arctic' APP mutation (E693G) causes Alzheimer's disease by enhanced Abeta protofibril formation. Nat Neurosci.

[B10] Chimon S, Shaibat MA, Jones CR, Calero DC, Aizezi B, Ishii Y (2007). Evidence of fibril-like β-sheet structures in a neurotoxic amyloid intermediate of Alzheimer's β-amyloid. Nat Struct Mol Biol.

[B11] Lührs T, Ritter C, Adrian M, Riek-Loher D, Bohrmann B, Döbeli H, Schubert D, Riek R (2005). 3D structure of Alzheimer's amyloid-β(1–42) fibrils. Proc Natl Acad Sci USA.

[B12] Petkova AT, Yau WM, Tycko R (2006). Experimental constraints on quaternary structure in Alzheimer's β-amyloid fibrils. Biochemistry.

[B13] Sørensen HP, Mortensen KK (2005). Advanced genetic strategies for recombinant protein expression in *Escherichia coli*. J Biotechnol.

[B14] Sharpe S, Yau WM, Tycko R (2005). Expression and purification of a recombinant peptide from the Alzheimer's β-amyloid protein for solid-state NMR. Protein Expr Purif.

[B15] Lopes DH, Colin C, Degaki TL, de Sousa AC, Vieira MN, Sebollela A, Martinez AM, Bloch C, Ferreira ST, Sogayar MC (2004). Amyloidogenicity and cytotoxicity of recombinant mature human islet amyloid polypeptide (rhIAPP). J Biol Chem.

[B16] Mazor Y, Gilead S, Benhar I, Gazit E (2002). Identification and characterization of a novel molecular-recognition and self-assembly domain within the islet amyloid polypeptide. J Mol Biol.

[B17] Döbeli H, Draeger N, Huber G, Jakob P, Schmidt D, Seilheimer B, Stuber D, Wipf B, Zulauf M (1995). A biotechnological method provides access to aggregation competent monomeric Alzheimer's 1–42 residue amyloid peptide. Biotechnology.

[B18] Hortschansky P, Schroeckh V, Christopeit T, Zandomeneghi G, Fändrich M (2005). The aggregation kinetics of Alzheimer's β-amyloid peptide is controlled by stochastic nucleation. Protein Sci.

[B19] Lee EK, Hwang JH, Shin DY, Kim DI, Yoo YJ (2005). Production of recombinant amyloid-β peptide 42 as an ubiquitin extension. Protein Expr Purif.

[B20] Shahnawaz M, Thapa A, Park IS (2007). Stable activity of a deubiquitylating enzyme (Usp2-cc) in the presence of high concentrations of urea and its application to purify aggregation-prone peptides. Biochem Biophys Res Commun.

[B21] Thapa A, Shahnawaz M, Karki P, Raj Dahal G, Golam Sharoar M, Yub Shin S, Sup Lee J, Cho B, Park IS (2008). Purification of inclusion body-forming peptides and proteins in soluble form by fusion to Escherichia coli thermostable proteins. Biotechniques.

[B22] Nagata-Uchiyama M, Yaguchi M, Hirano Y, Ueda T (2007). Expression and purification of uniformly ^15^N-labeled amyloid β peptide 1–40 in *Escherichia coli*. Protein Pept Lett.

[B23] Tolia NH, Joshua-Tor L (2006). Strategies for protein coexpression in Escherichia coli. Nat Methods.

[B24] Henricksen LA, Umbricht CB, Wold MS (1994). Recombinant replication protein A: expression, complex formation, and functional characterization. J Biol Chem.

[B25] Li C, Schwabe JW, Banayo E, Evans RM (1997). Coexpression of nuclear receptor partners increases their solubility and biological activities. Proc Natl Acad Sci USA.

[B26] Romier C, Ben Jelloul M, Albeck S, Buchwald G, Busso D, Celie PH, Christodoulou E, De Marco V, van Gerwen S, Knipscheer P (2006). Co-expression of protein complexes in prokaryotic and eukaryotic hosts: experimental procedures, database tracking and case studies. Acta Crystallogr D.

[B27] Stebbins CE, Kaelin WG, Pavletich NP (1999). Structure of the VHL-ElonginC-ElonginB complex: implications for VHL tumor suppressor function. Science.

[B28] Schlieker C, Bukau B, Mogk A (2002). Prevention and reversion of protein aggregation by molecular chaperones in the *E. coli *cytosol: implications for their applicability in biotechnology. J Biotechnol.

[B29] Thomas JG, Ayling A, Baneyx F (1997). Molecular chaperones, folding catalysts, and the recovery of active recombinant proteins from *E. coli*. Appl Biochem Biotechnol.

[B30] Grönwall C, Jonsson A, Lindstrom S, Gunneriusson E, Ståhl S, Herne N (2007). Selection and characterization of Affibody ligands binding to Alzheimer amyloid β peptides. J Biotechnol.

[B31] Nygren PÅ (2008). Alternative binding proteins: Affibody binding proteins developed from a small three-helix bundle scaffold. FEBS J.

[B32] Nord K, Gunneriusson E, Ringdahl J, Ståhl S, Uhlén M, Nygren PÅ (1997). Binding proteins selected from combinatorial libraries of an a-helical bacterial receptor domain. Nat Biotechnol.

[B33] Hoyer W, Grönwall C, Jonsson A, Ståhl S, Härd T (2008). Stabilization of a β-hairpin in monomeric Alzheimer's amyloid-β peptide inhibits amyloid formation. Proc Natl Acad Sci USA.

[B34] Gardberg AS, Dice LT, Ou S, Rich RL, Helmbrecht E, Ko J, Wetzel R, Myszka DG, Patterson PH, Dealwis C (2007). Molecular basis for passive immunotherapy of Alzheimer's disease. Proc Natl Acad Sci USA.

[B35] Hou L, Shao H, Zhang Y, Li H, Menon NK, Neuhaus EB, Brewer JM, Byeon IJ, Ray DG, Vitek MP (2004). Solution NMR studies of the Aβ(1–40) and Aβ(1–42) peptides establish that the Met35 oxidation state affects the mechanism of amyloid formation. J Am Chem Soc.

[B36] Hepler RW, Grimm KM, Nahas DD, Breese R, Dodson EC, Acton P, Keller PM, Yeager M, Wang H, Shughrue P (2006). Solution state characterization of amyloid β-derived diffusible ligands. Biochemistry.

[B37] Walsh DM, Lomakin A, Benedek GB, Condron MM, Teplow DB (1997). Amyloid beta-protein fibrillogenesis. Detection of a protofibrillar intermediate. J Biol Chem.

[B38] Fezoui Y, Hartley DM, Harper JD, Khurana R, Walsh DM, Condron MM, Selkoe DJ, Lansbury PT, Fink AL, Teplow DB (2000). An improved method of preparing the amyloid β-protein for fibrillogenesis and neurotoxicity experiments. Amyloid.

[B39] Terzi E, Holzemann G, Seelig J (1995). Self-association of β-amyloid peptide (1–40) in solution and binding to lipid membranes. J Mol Biol.

[B40] LeVine H (1999). Quantification of β-sheet amyloid fibril structures with thioflavin T. Methods Enzymol.

[B41] Wang H, Chong S (2003). Visualization of coupled protein folding and binding in bacteria and purification of the heterodimeric complex. Proc Natl Acad Sci USA.

[B42] Hoyer W, Härd T (2008). Interaction of Alzheimer's Aβ peptide with an engineered binding protein – thermodynamics and kinetics of coupled folding-binding. J Mol Biol.

[B43] Binz HK, Amstutz P, Plückthun A (2005). Engineering novel binding proteins from nonimmunoglobulin domains. Nat Biotechnol.

[B44] Wiesehan K, Funke SA, Fries M, Willbold D (2007). Purification of recombinantly expressed and cytotoxic human amyloid-beta peptide 1–42. J Chromatogr B.

[B45] Gorman PM, Chakrabartty A (2001). Alzheimer beta-amyloid peptides: structures of amyloid fibrils and alternate aggregation products. Biopolymers.

[B46] Stine WB, Dahlgren KN, Krafft GA, LaDu MJ (2003). In vitro characterization of conditions for amyloid-beta peptide oligomerization and fibrillogenesis. J Biol Chem.

[B47] Carrotta R, Di Carlo M, Manno M, Montana G, Picone P, Romancino D, San Biagio PL (2006). Toxicity of recombinant β-amyloid prefibrillar oligomers on the morphogenesis of the sea urchin Paracentrotus lividus. FASEB J.

[B48] Lesley SA (2001). High-throughput proteomics: protein expression and purification in the postgenomic world. Protein Expr Purif.

[B49] Thiel MA, Coster DJ, Mavrangelos C, Zola H, Williams KA (2002). An economical 20 litre bench-top fermenter. Protein Expr Purif.

[B50] Meinhardt J, Tartaglia GG, Pawar A, Christopeit T, Hortschansky P, Schroeckh V, Dobson CM, Vendruscolo M, Fändrich M (2007). Similarities in the thermodynamics and kinetics of aggregation of disease-related Aβ(1–40) peptides. Protein Sci.

[B51] Peim A, Hortschansky P, Christopeit T, Schroeckh V, Richter W, Fändrich M (2006). Mutagenic exploration of the cross-seeding and fibrillation propensity of Alzheimer's β-amyloid peptide variants. Protein Sci.

[B52] Hou L, Zagorski MG (2006). NMR reveals anomalous copper(II) binding to the amyloid Aβ peptide of Alzheimer's disease. J Am Chem Soc.

[B53] Grant MA, Lazo ND, Lomakin A, Condron MM, Arai H, Yamin G, Rigby AC, Teplow DB (2007). Familial Alzheimer's disease mutations alter the stability of the amyloid β-protein monomer folding nucleus. Proc Natl Acad Sci USA.

[B54] Huang TH, Yang DS, Plaskos NP, Go S, Yip CM, Fraser PE, Chakrabartty A (2000). Structural studies of soluble oligomers of the Alzheimer β-amyloid peptide. J Mol Biol.

[B55] Delaglio F, Grzesiek S, Vuister GW, Zhu G, Pfeifer J, Bax A (1995). NMRpipe: A multidimensional spectral processing system based on UNIX pipes. J Biomol NMR.

[B56] Vranken WF, Boucher W, Stevens TJ, Fogh RH, Pajon A, Llinas M, Ulrich EL, Markley JL, Ionides J, Laue ED (2005). The CCPN data model for NMR spectroscopy: development of a software pipeline. Proteins.

